# Bis-class: a new classification tool of methylation status using bayes classifier and local methylation information

**DOI:** 10.1186/1471-2164-15-608

**Published:** 2014-07-18

**Authors:** Iksoo Huh, Xingyu Yang, Taesung Park, Soojin V Yi

**Affiliations:** Department of Statistics, Bioinformatics and Biostatistics Laboratory, Seoul National University, 151-742 Seoul, Korea; School of Biology, Institute of Bioengineering and Biosciences, Georgia Institute of Technology, 310 Ferst Drive, 30332 Atlanta, GA USA

**Keywords:** DNA methylation, Bayes classifier, Local DNA methylation level, MethylC-seq

## Abstract

**Background:**

Whole genome sequencing of bisulfite converted DNA (‘methylC-seq’) method provides comprehensive information of DNA methylation. An important application of these whole genome methylation maps is classifying each position as a methylated versus non-methylated nucleotide. A widely used current method for this purpose, the so-called binomial method, is intuitive and straightforward, but lacks power when the sequence coverage and the genome-wide methylation level are low. These problems present a particular challenge when analyzing sparsely methylated genomes, such as those of many invertebrates and plants.

**Results:**

We demonstrate that the number of sequence reads per position from methylC-seq data displays a large variance and can be modeled as a shifted negative binomial distribution. We also show that DNA methylation levels of adjacent CpG sites are correlated, and this similarity in local DNA methylation levels extends several kilobases. Taking these observations into account, we propose a new method based on Bayesian classification to infer DNA methylation status while considering the neighborhood DNA methylation levels of a specific site. We show that our approach has higher sensitivity and better classification performance than the binomial method via multiple analyses, including computational simulations, Area Under Curve (AUC) analyses, and improved consistencies across biological replicates. This method is especially advantageous in the analyses of sparsely methylated genomes with low coverage.

**Conclusions:**

Our method improves the existing binomial method for binary methylation calls by utilizing a posterior odds framework and incorporating local methylation information. This method should be widely applicable to the analyses of methylC-seq data from diverse sparsely methylated genomes. Bis-Class and example data are provided at a dedicated website (http://bibs.snu.ac.kr/software/Bisclass).

**Electronic supplementary material:**

The online version of this article (doi:10.1186/1471-2164-15-608) contains supplementary material, which is available to authorized users.

## Background

DNA methylation is a prevalent epigenetic modification of genomic DNA with fundamental functional consequences on developmental processes, regulation of gene expression and diseases [1, 2]. Accurately inferring the level of DNA methylation at a specific nucleotide in the genome is a critical step toward elucidating the molecular mechanisms of regulation via DNA methylation. A method widely gaining popularity is the whole genome sequencing of bisulfite converted genomic DNA, often referred to as ‘methylC-seq’ (also referred to as ‘BS-seq’ elsewhere). This method is based upon the particular chemical properties of DNA methylation to ‘protect’ cytosines from converting to uracils by sodium bisulfite [3]. Specifically, during the sodium bisulfite conversion process, non-methylated cytosines are changed to uracils, which then change to thymine after PCR amplification. Consequently, following a sodium bisulfite treatment, non-methylated cytosines should be read as thymines while methylated cytosines should remain as cytosines.

Compared to microarray-based methods, the methylC-seq method is powerful in a multitude of ways. In addition to the fact that it can provide information on every nucleotide in the genome, a notable strength of this method is that it can be applied to analyses of non-model species where pre-defined microarrays (such as beadchip) are not readily available. For this reason, methylC-seq has been instrumental in the recent surge of genomic DNA methylation analyses from diverse taxa, in particular from many invertebrates (e.g., [4–6]). These studies show that invertebrate genomes generally exhibit very different patterns of DNA methylation compared to those of mammalian genomes. The most significant difference is that invertebrate genomes tend to be much more sparsely methylated than mammalian genomes. For example, the mean level of DNA methylation for individual CpGs in the honey bee genome is <1% [7, 8], which is far lower than that in the human genome (60 ~ 90% [9, 10]). Even relatively heavily methylated genomes of some aquatic species such as the freshwater snail *Biomphalaria glabrata* or the pacific oyster *Crassostrea gigas* harbor only a few percent of methylated cytosines [11]. Similarly, plant genomes appear to be generally much more sparsely methylated than mammalian genomes. For example, only a few percent of cytosines are methylated during the early stages of *Populous* floral development [12].

**Figure 1:**
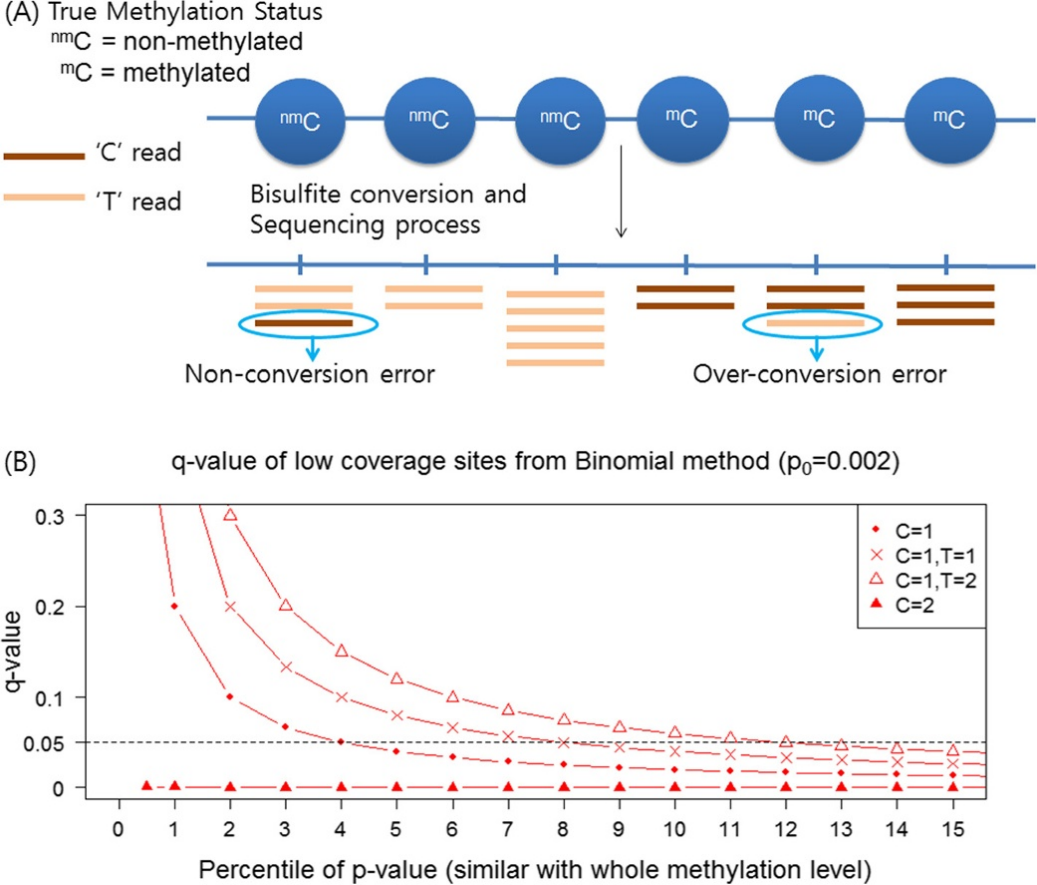
**Potential errors and biases of methylC-seq and binomial method.** **(A)** Errors associated with the methylC-seq method. Non-methylated Cs may not be completely converted (non-conversion error, non-methylated C remains as C). In addition, methylated Cs may undergo conversion (over-conversion error, methylated C converts to T). **(B)** Reduced power of the binomial test in sparsely methylated genomes and low coverage. The Y-axis indicates FDR-corrected q-values from the binomial test, calculated following the equation (2) in the main text. The X-axis indicates the percentiles of p-values, which is equivalent to the whole genome methylation levels. Four cases are shown, including when a specific cytosine is covered by a single ‘C’ read (filled circles), one ‘C’ and one ‘T’ reads (crosses), one ‘C’ and two ‘T’ reads (open triangles) and two ‘C’ reads (filled triangles). The fractional methylation levels of these four cases are all substantial, 100%, 50%, 33% and 100%, respectively. However, in sparsely methylated genomes, many of these sites will have q-value > 0.05 and will be classified as ‘unmethylated’. For example, when only a single ‘C’ read is available (line with filled circles), despite the fact that the read itself indicates a 100% methylation, it will be designated as unmethylated (q-values > 0.05) unless the overall methylation level of the genome is greater than 4%. In another case, when a C is covered by one ‘C’ Read and two ‘T reads (line with open triangles), the fractional methylation level of such a position is 33%. However, such a site will be called as ‘unmethylated’ unless the overall level of methylation in the genome is 12% or higher.

Analyzing such sparsely methylated genomes presents unique technical challenges. In heavily methylated species such as mammals, the measure of interest is usually the fraction of methylated reads (‘C’ reads) in the total number of reads per site, the so-called ‘fractional DNA methylation’ [13–15]. In sparsely methylated genomes, these values are typically very small. Moreover, these values are heavily influenced by errors associated with the conversion and sequencing processes (see below). For these reasons, it is often important to determine whether a specific position has any methylation or not. In other words, a *binary* classification of methylated versus non-methylated cytosines is critical to evaluate the distribution of DNA methylation and different levels of DNA methylation [4, 8, 16, 17]. In principle, this should be simple: cytosines covered by *any* number of ‘C’ reads should be considered methylated. However, in reality, this step is not straightforward due to the nature of chemistry underlying the MethylC-seq method. Specifically, the sodium bisulfite conversion step is not perfect, and includes both i) the possibility of non-conversion (non-methylated C is not properly converted to U/T), leading to an *over*estimation of actual DNA methylation (Figure 1A), as well as ii) over-conversion (methylated C is also converted), leading to an *under*estimation of actual DNA methylation (Figure 1A) [3]. Consequently it is necessary to take into account these technical errors for a binary classification of a specific nucleotide. In particular, these errors can occur at rates comparable to the actual methylation levels in some genomes. Despite these well-known and substantial technical issues, methods to efficiently account for these imperfections are surprisingly rare. The most widely used method is the so-called *binomial method* [8, 17]. However, this method has some shortcomings when the genomic methylation levels and the coverage of specific site are low (see below).

**Figure 2:**
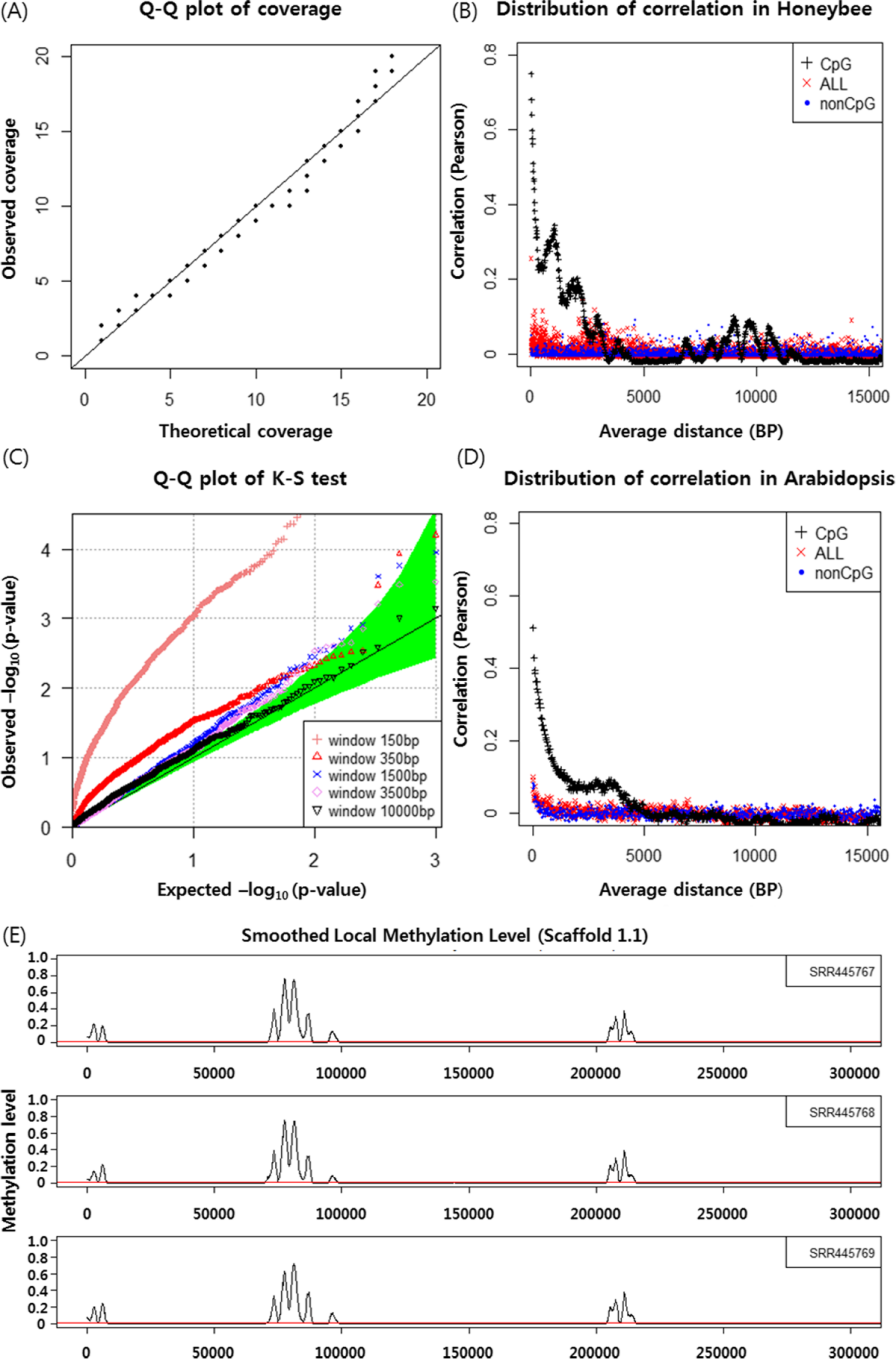
**Properties of methylC**-**seq coverage and spatial correlation of CpG methylation level.** **(A)** Quantile-quantile (Q-Q) plot between observed coverage and theoretical coverage which is from a shifted negative binomial distribution. **(B)** Spatial correlation plot of a honeybee methylome from Herb et al. [7]. **(C)** Q-Q plot between observed p-values from Kolmogorov-Smirnov (K-S) test and theoretical p-values from a null distribution. The K-S test was used to detect the spatial correlation between selected CpG sets, consisting of one CpG per window. Detailed procedure is explained in the Additional file 1. The green region implies 95% confidence interval of theoretical ordered p-values. The overlap between the observed P-values indicates that the data follows the null distribution. **(D)** Spatial correlation plot of an *Arabidopsis* methylome (methylC-seq data from GSM276809, [29]). **(E)** Smoothed methylation level using triangle kernel in scaffold 1.1, for three samples. X-axis is physical location and Y-axis is methylation level. Red lines represent average methylation fractions calculated from whole CpG methylomes.

Here, we propose a new method, the Bisulfite-sequencing data classification method (Bis-Class). This method takes the prior methylation distribution into account to infer methylation status in the framework of Bayesian probabilistic models, which is known to minimize classification errors in the presence of a known alternative hypothesis [18]. In addition to utilizing a Bayesian classification scheme, we take into account the fact that DNA methylation levels of adjacent sites are correlated (Figure 2). Consequently, including information on DNA methylation levels of the genomic neighborhood improves our ability to correctly infer the DNA methylation status. We demonstrate that Bis-Class alleviates the problems of the binomial method and improve sensitivity and accuracy using extensive simulations as well as analyses of actual methylC-seq data.

## Methods

### Pitfalls of the binomial method

We first describe the widely used binomial method in some detail. In this method, the probability that a non-methylated C remains as C, or the ‘non-conversion’ error (which we will refer to as *p*_*0*_), is used to infer whether the observed methylation signal is more likely to have arisen by chance. Specifically, the methylation status of a site is determined under a binomial distribution where *p*_*0*_ is used as the success rate. The null hypothesis is that the site is not methylated, and the *P*-value for this null hypothesis is:
1

where *k* is the number of methylated reads at the site of interest, and *N* is the total number of reads at this site. The resulting *P*-values are further corrected for multiple testing, typically by the false discovery rate (FDR) [19]. The main parameter *p*_*0*_ is determined either by examining non-methylated portions of the genome (such as repetitive regions in insect genomes or chloroplast genomes in plants, e.g., [8, 17]) or from ‘spiked-in’ lambda genomic DNA (e.g., [13]). This approach is intuitive and straightforward.

However, the power of the binomial method is weak when the number of reads (*N*) is small (i.e., equation (1) above). Moreover, when combined with the correction for multiple testing with FDR, the power of the binomial method is particularly reduced at low coverage sites in sparsely methylated genomes. This reduction is because the *FDR*-corrected *q*-value of the *i*^*th*^ site is calculated as
2

where *p*_*i*_ is the binomial *P*-value for a specific site *i*. Since the binomial *P*-values are limited by the number of reads (*N*) for that site, *P*-values from low-coverage sites (low *N*) will have moderate ranks at most. Consequently, in sparsely methylated genomes, even if a site is truly methylated, if the number of reads is small, the *P*-value of such site will not be ranked sufficiently low to be classified as methylated after FDR correction. Figure 1B demonstrates this phenomenon using specific examples. For example, a site covered by a single ‘C’ read (the line with filled circles, Figure 1B) will not be classified as ‘methylated’ with the binomial method in genomes with the overall methylation levels typical of hymenopteran insects (i.e., < 4%). Likewise, a site with 50% methylation with coverage of two (the line with crosses, Figure 1B) will also be classified as non-methylated in sparsely methylated genomes. Consequently, the binomial method may produce a high number of false negatives in lowly methylated genomes. To avoid this problem, some studies suggest using sites that are covered by at least two [20], or four [17] reads. However, since the number of reads typically has a large variance, even with a moderate coverage sequence data, substantial numbers of cytosines are covered by single reads (Table 1), making it impractical to remove positions with a small number of reads. For example, if we remove sites with fewer than four reads, almost 50% of data are discarded in representative methylC-seq datasets in honey bee (Table 1).

**Table 1:** **Properties of the methylC**-**seq data sets used in this study**

Species	Subtype	Sample ID	Coverage	Variance	Variance/Coverage	Proportion of under 3 reads (% of 1 read)
Honey Bee	**Worker**	SRR039814	5.86	22.96	3.918	0.4255 (0.1547)
	**Queen**	SRR039815	7.24	27.521	3.801	0.3728 (0.1118)
	**Forager**	SRR445767	3.86	10.28	2.663	0.5608 (0.1892)
		SRR445768	4.17	13.27	3.182	0.5345 (0.1825)
		SRR445769	3.86	9.951	2.578	0.5620 (0.1944)
		SRR445770	4.04	12.838	3.177	0.5521 (0.1907)
		SRR445771	4.51	14.510	3.217	0.4823 (0.1522)
		SRR445773	5.86	18.275	3.119	0.3111 (0.0798)
	**Nurse**	SRR445774	3.13	6.081	1.943	0.6709 (0.2512)
		SRR445775	4.49	14.812	3.299	0.4868 (0.1552)
		SRR445776	3.84	12.203	3.178	0.5802 (0.2032)
		SRR445777	4.51	14.978	3.321	0.4856 (0.1553)
		SRR445778	2.65	6.846	2.583	0.7801 (0.359)
		SRR445799	4.05	12.014	2.966	0.5434 (0.1875)
Human (Brain)		HS1570_0731	8.59	37.157	4.32	0.2874 (0.0945)

### Bayes classification using methylation level as prior probabilities

To overcome the aforementioned problems in the binomial method, here we propose to use a Bayesian probabilistic model to infer methylation status. The posterior probability of methylation status is determined based upon the product of prior distribution of methylation and the likelihood of specific reads aligned to a site. Specifically, the posterior distribution of methylation is given as.

, where *M* is a random variable representing methylation status (*m* for methylated, *nm* for non-methylated). R = {R_1_, R_2,..,_ R_N_} is the set of sequence reads mapped to a site. If a sample consists of *N* number of CpGs and *i*^*th*^ CpG has *n*_*i*_ reads, *R*_*i*_ denotes a set of reads assigned in *i*^*th*^ CpG and *R*_*ij*_ denotes *j*^*th*^ read of *i*^*th*^ CpG (*i* = 1, …, *N* and *j* =1, …, *n*_*i*_). In addition, likelihood *P* (*R*_*i*_|*M*) is given as the product of *P* (*R*_*ij*_|*M*) s. *π* (*M*) is the prior information on DNA methylation.

### Derivation of *P*(*R*|*M*)

The likelihood *P* (*R*_*i*_|*M*) can be calculated by explicitly incorporating the errors associated with the inference of methylation. The main source of errors for non-methylated sites is the non-conversion rate (denoted as *p*_*0*_, Figure 1A). If there is no additional error, the probability of obtaining a C read in non-methylated site is equivalent to the non-conversion rate *p*_*0*_. Likewise, the probability of obtaining a C read in methylated site is 1- (over-conversion rate), which we denote as *p*_*1*_ (Figure 1A). There may be additional errors occurring during sequencing process. We define the sequencing error (ϵ) as the probability of being misread from other nucleotide (For example, reading C read as T read or vice versa).

Consequently, our observation likelihood *P* (*R*_*i*_|*M*) consists of the following distributions according to the methylation status.
34

Since sequencing errors are confounded with *p*_*0*_ or *p*_*1*_ in reality, we will regard *p*_*0*_′ and *p*_*1*_′ as *p*_*0*_ and *p*_*1*_ in this article, respectively. The parameters *p*_*0*_ or *p*_*1*_ are inferred from data using the Expectation-Maximization (EM) algorithm [21]. The details of this calculation are shown in the Additional file 1.

### Incorporating local methylation information to improve inference

We demonstrate below that methylated cytosines are heterogeneously distributed and locally clustered in different species (Figure 2). For example in the honeybee genome, some regions exhibit >70-fold higher methylation levels compared to other regions (Figure 2E). We take this observation into account and incorporate local methylation levels into the methylation prior to improve classification accuracy. Since methylated cytosines are heterogeneously distributed and locally clustered, the use of local methylation information would be useful. Since some regions may have extreme methylation values, it might be also useful to include information on the global methylation level. Here, we propose using a weighted average of local and global methylation levels to produce a more robust estimation of posterior odds. Specifically, if we denote the global methylation level as *π*_1_^*G*^ and the local methylation level as *π*_1_^*L*^, the combined methylated prior, *π*_1_^*C*^, can be represented as *π*_1_^*G*^ × (1 - *w*) + *π*_1_^*L*^ × *w*. The weight parameter, *w*, can decide how much local versus global methylation levels can be included in the prior. This factor can have any value between 0 and 1. In our analyses we used 0.5, to treat local and global information equally. In our experience, using the weight factor of 0.5 produced good AUC (Area Under Curve), sensitivity and low error rate compared with other weight factor values for honey bee data (Additional file 2). Nevertheless, in this implementation of Bis-Class the users can choose any arbitrary value of the weight factor.

The global methylation level can be estimated from the observed numbers of C and T reads, as an extension of the widely used ‘fractional methylation levels’ [13–15]. Details are presented in the Additional file 1. In order to estimate local methylation levels (denoted as ), we additionally use the kernel function which adjusts the weight of a specific function to consider distance from the site which is to be determined. For a kernel function *K* (*d*), *d* is the physical distance from a site which is of interest. Then  can be estimated as weighted average through kernel function, as follows:
5

 and  are estimates of *p*_0_ and *p*_1_, respectively. *L* is the number of reads for a specific site, and *F* is the number of ‘C’ reads divided by the total number of reads (Additional file 1), and is equivalent to the commonly used ‘fractional methylation’ measures [13–15]. *k* = 1, 2, …, K denotes the index of CpGs in a window. The kernel function *K* (*d*) can be many types of functions which decreases as *d* increases. In our analyses, we chose to use the triangle kernel which decreases linearly for *d* ≤ *d*_*0*_ and zero for *d* > *d*_*0*_. In addition, we define a window around the considered site as the region whose kernel weights are not zero. The window size, *d*_*0*_, can be arbitrarily selected. We also define *K* (*0*) = 0 to exclude the focal cytosine. Our approach is very flexible, as the width and the kernel type can be easily changed according to the properties of each dataset. We selected the triangle kernel because it is similar to be observed patterns of spatial correlation between methylation levels of adjacent CpG sites (Figure 2B). Applying alternative kernels such as Gaussian or Laplace provided similar results (Additional file 2). The width of kernel in our analyses was determined as the point where the spatial correlation decreases to below 0.2, which is approximately 1.5 kb in the honey bee data (Figure 2B).

### Posterior odds

After following the above steps, the posterior odds for *i*^*th*^ CpG can be constructed as:
6

If the value of a specific site is larger than some criteria, it will be classified as methylated. We propose using 19 as the criterion (meaning that the probability of being classified as methylated is 19 times bigger than that of being classified as non-methylated). This criterion also means that the probability of being falsely classified as methylated is smaller than 0.05 at the site [22]. Consequently this is equivalent to the FDR-corrected q-value < 0.05, as typically used in the binomial test.

## Results and discussion

### Features of MethylC-seq data with emphasis on honey Bee

In this section we present analyses of actual bisulfite-sequencing data that are pertinent to our proposed method. Honey bee is one of the first invertebrates for which the methylC-seq method has been applied. The usage of the methylC-seq method has been crucial to elucidating the importance of DNA methylation on gene regulation in honey bee, including its role in the differentiation of castes [8], behavioral differentiation of worker bees [7], and alternative splicing [23, 24]. We examined two recent methylC-seq datasets of honey bee, one from the brains of worker and queen bees [8], the other from brains of six forager and six nurse bees [7]. All data have been mapped to the assembly 2.0 using BSmap [25].

As reported previously in the original studies, mean fractional methylation levels are extremely low, between 0.3 ~ 0.5% for all cytosines, and 0.5% ~ 0.9% for CpGs ( in Table 2). The mean coverage in these data sets ranges between 2.65 and 7.24 (Table 1) and the variance of read depths is quite high (Table 1). The distribution of coverage follows a shifted negative binomial distribution with similar mean and variance as observed (Figure 2A). An important consequence of this is that most of the data (~50%) are covered by fewer than four reads and a substantial portion of the data are covered by only a single read (Table 1).

**Table 2:** **Methylation classification using the binomial and Bis**-**Class methods**

Species	Subtype	Sample ID	(experiment)	(EM)	(EM)	(CpG only)	(CpG only)	(***p***_1_=0.95) (CpG only)	Count of mCpG (binomial)	Count of mCpG (Bis-Class)
**Honey Bee**	**Worker**	SRR039814	0.0029	0.0021	0.7081	0.004985 (0.005414)	0.004086 (0.004694)	0.002727 (0.003496)	83509	111900
	**Queen**	SRR039815	0.0024	0.0018	0.7273	0.004009 (0.005750)	0.003044 (0.005472)	0.00232 (0.003872)	98769	117829
	**Forager**	SRR445767	0.0012	0.0015	0.6669	0.003271 (0.007931)	0.002662 (0.009665)	0.001867 (0.006780)	97220	106204
		SRR445768	0.0013	0.0015	0.6479	0.003309 (0.008171)	0.002799 (0.01032)	0.001907 (0.007033)	101628	108382
		SRR445769	0.0011	0.0014	0.6608	0.0032 (0.007830)	0.002730 (0.009752)	0.001897 (0.006778)	96934	105128
		SRR445770	0.0012	0.0014	0.6461	0.00322 (0.008059)	0.002823 (0.01033)	0.001918 (0.007019)	100173	107755
		SRR445771	0.0012	0.0014	0.6682	0.003269 (0.008434)	0.002803 (0.01055)	0.001970 (0.007415)	107688	111434
		SRR445773	0.0013	0.0014	0.6574	0.003087 (0.007448)	0.002572 (0.009221)	0.001778 (0.006375)	117971	116825
	**Nurse**	SRR445774	0.0012	0.0015	0.6888	0.003115 (0.007391)	0.002350 (0.008571)	0.001702 (0.006201)	75340	95191
		SRR445775	0.0011	0.0015	0.6539	0.003209 (0.007849)	0.002620 (0.009733)	0.001801 (0.006693)	103257	109181
		SRR445776	0.0012	0.0015	0.6506	0.003113 (0.007717)	0.002485 (0.009579)	0.001700 (0.006554)	92131	103492
		SRR445777	0.0011	0.0015	0.6571	0.003228 (0.007936)	0.002636 (0.009818)	0.001821 (0.006785)	105435	110196
		SRR445778	0.0013	0.0013	0.6563	0.003252 (0.008262)	0.002980 (0.01063)	0.002057 (0.007338)	63375	90489
		SRR445799	0.0012	0.0015	0.6574	0.003314 (0.008137)	0.002766 (0.01012)	0.001912 (0.006997)	99063	106836
**Human** **(Brain)**		HS1570_0731	0.0013	0.01	0.94	(0.8064)	(0.8563)	(0.8472)	901228^a^	882351^a^

^a^Calling only on 10^6^ CpGs in Chr 10.

### Correlated levels of local DNA methylation

Methylated cytosines are not randomly distributed along the genome. DNA methylation levels of nearby cytosines are correlated; for example, in a forager sample from Herb et al. [7], the correlation coefficient between two CpGs 100 bps apart is 0.5 (Figure 2B). The correlation decreases as the distance between two cytosines increases, and this pattern is more pronounced for CpGs than non-CpGs (Figure 2B). Co-variation of DNA methylation of adjacent cytosines extends to several kilobases (Figure 2B and 2C). We observed similar trends in multiple species analyzed. For example, in *Arabidopsis*, a similar pattern is observed (Figure 2D, also see [26]). A similar level of spatial correlation has been also observed in the human genome [27]. When examined in detail, methylated cytosines in the honey bee are locally clustered in the genome (Figure 2E), with several regions in the chromosome exhibiting elevated levels of DNA methylation (Figure 2E). Importantly, this pattern and the locations of methylated clusters are consistent across different biological replicates (Figure 2E), indicating that the spatial correlation is not caused by technical noises, but reflects the inherent pattern in the genomic distributions of DNA methylation in these species.Together with the results in the above section, we show that a substantial portion of the genome is covered by very few reads, the overall level of methylation is low, and that local methylation levels are correlated. As discussed above and seen in the Figure 1, such aspects of data render the binomial method prone to high false negative rates. Consequently, we propose Bis-Class as a practical alternative to the commonly used binomial method of classification. In the next section, we show comprehensive simulation results based upon the observed parameters of the data, indicating that Bis-Class outperforms the binomial method.

### Improved sensitivity and accuracy of methylation calling by Bis-class

We performed extensive simulation to compare the performance of Bis-Class to the binomial method. We generated methylC-seq data for a genome of 100,000 cytosines, with the mean coverage ranging between 3× to 9×. The numbers of total reads at each site were generated from a shifted negative binomial distribution with the whole genome coverage as the mean and three times the mean as the variance, similar to the typical methylC-seq dataset in honey bee (Table 1, Figure 2A). The selected parameters *p*_*0*_ and *p*_*1*_, as well as the total methylation levels are also similar to those observed in the empirical data (Table 2). We also examined the effects of each parameter when they were slightly greater than the observed values. The weight parameter we used is 0.5, to consider global information and local information equally. To examine the effect of DNA methylation clustering, we generated two types of genomes. The first is a genome where methylated CpGs are uniformly distributed. In the second type, DNA methylation is concentrated in 1/10 of the genome in a 10× intensity compared to whole genome methylation level. We generated 100 replicates for each parameter combination. Local information was obtained from the 200 nearest cytosines (which is equivalent to considering CpGs with 3000 bps of a specific site in the honey bee methylation data).

**Figure 3:**
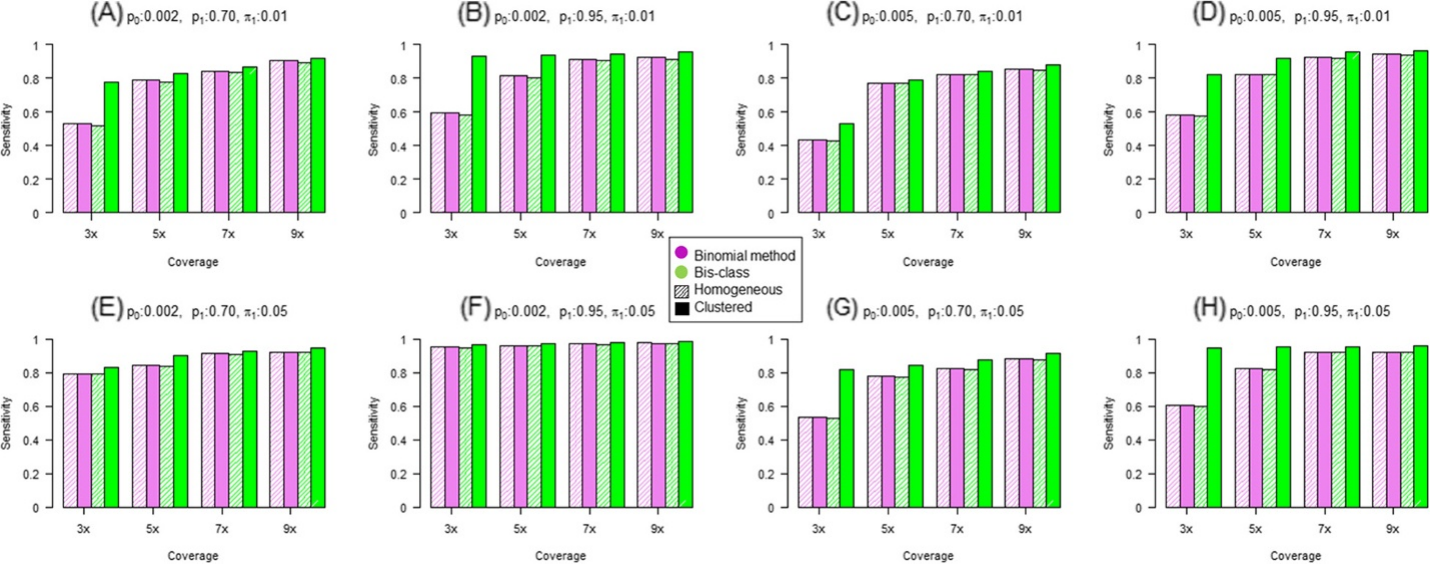
**Comparison of sensitivities of Bis-** **Class and the binomial method using simulated data.** Sensitivities are evaluated in a variety of parameter settings and plotted in **(A)**-**(H)**. Purple bars and green bars indicate the results from the binomial method and the Bis-Class, respectively. Bars with diagonal lines indicate the results from homogeneous methylomes and solid bars indicate those from regionally clustered methylomes.

**Figure 4:**
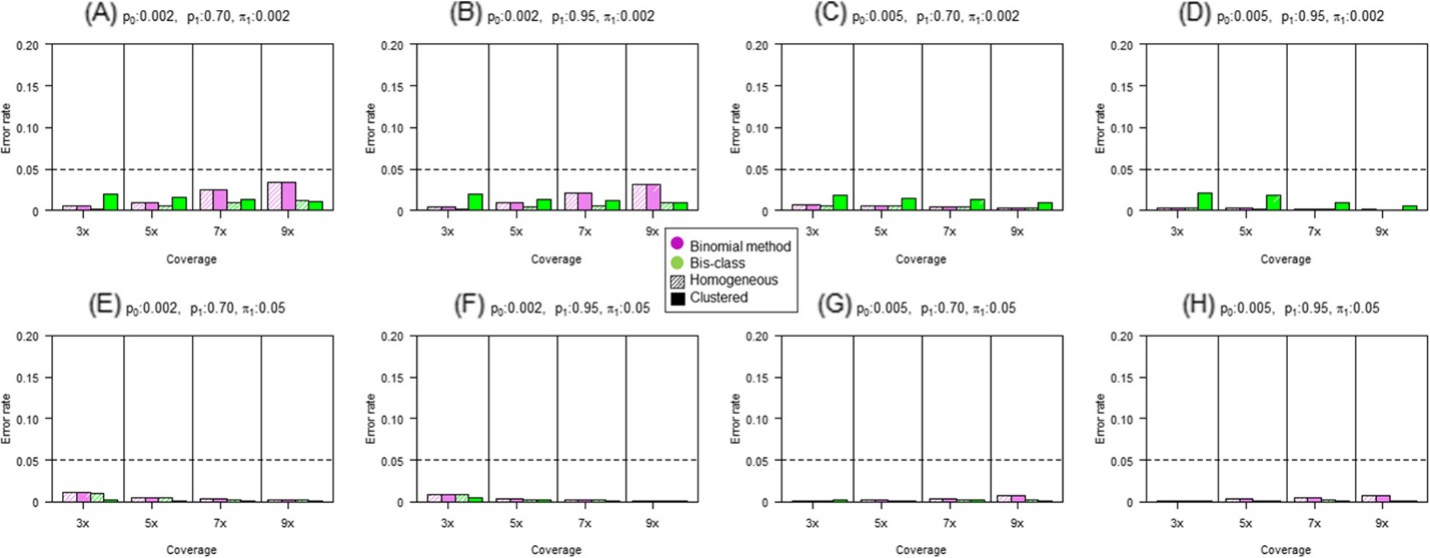
**Comparison of misclassification rates of non**-**methylated CpGs via the Bis**-**Class and the binomial method using simulated data.** 1-specificities are evaluated in a variety of parameter settings and plotted in **(A)**-**(H)**. The Y-axis indicates the ratio of the number of misclassified non-methylated CpGs to the total number of methylated CpGs. Purple bars and green bars are the results from the binomial method and the Bis-Class, respectively. Bars with diagonal lines imply the results from homogeneous methylomes and solid bars imply those from regionally clustered methylomes.

We then compared classification results with the true status and calculated the sensitivity as the proportion of sites classified as methylated when they are truly methylated (Figure 3). The higher the sensitivity, the lower the rate of false negatives. In genomes where DNA methylation occurs uniformly (‘homogeneous’), both the binomial method and Bis-Class provide similar results across almost all settings (purple and green bars filled with diagonal lines in Figure 3). We note that the binomial methods in clustered genomes and homogenous genomes are statistically equivalent, which is apparent in the simulation results. Sensitivities are low when the sequence coverage is low, and increase with sequence coverage. In the non-homogenous, clustered genomes, Bis-Class (solid green bar) outperforms the binomial method and exhibit much higher sensitivity (therefore lower false negatives) than the binomial method (solid purple bar, Figure 3). While Bis-Class displays higher sensitivities compared to the binomial method in a variety of settings, the difference is most pronounced when the coverage is low. In addition, the difference between Bis-Class and the binomial method is large when the ratios between the two error rates (*p*_*0*_ and *p*_*1*_) are high and the whole genome methylation level is low.We also examined the incidences of mis-classification. Because the proportions of methylation and non-methylation sites are not balanced, a direct comparison between accuracy measures is difficult to perform. For this, we define ‘1-specificity’ as the ratio of the number of mis-classified non-methylated sites to the number of true methylated sites. The resulting plots (Figure 4) show that all methods have acceptably low error rates (less than five percent of true methylated sites).

These simulation results demonstrate that, with the cutoff comparable to FDR-corrected q-value < 0.05, Bis-Class exhibits a greater sensitivity and a comparable specificity compared to the binomial method. Overall, Bis-Class has a greater accuracy (calculated as the sum of (proportion of methylated sites) x sensitivity and (proportion of non-methylated sites) x specificity) than the binomial method. To illustrate this further we evaluated the Area Under Curve (AUC) measure of the ROC (Receiver operating characteristic) under identical simulation settings, which is expected to provide a comprehensive comparison because it summarizes both sensitivity and specificity across all possible cutoff values [28]. This analysis (Additional file 3) demonstrates that the AUC values of Bis-Class are larger than those of the binomial method, especially in settings where the sequence coverage is low and DNA methylation occurs heterogeneously, i.e., settings closely resembling the observed patterns in the actual methylC-seq data (Tables 1 and 2, Figure 2). Together these results indicate that Bis-Class provides superior results compared to the binomial method.

### Application of Bis-class to MethylC-Seq data

We applied the Bis-Class to the aforementioned honey bee data sets. We first estimated the parameters *p*_*0*_ and *p*_*1*_ using the EM algorithm. The results are shown in Table 2; all data had very low *p*_*0*_, indicating that the error rates due to non-conversion are small. Importantly, the *p*_*0*_ estimated from EM are highly similar to the values provided by the authors using experimental methods (Table 2). The estimates of *p*_*1*_ values are around 70% for honey bee data sets. These are much lower than the estimate from the human genome (Table 2). The underlying cause for this discrepancy needs to be studied in future experiments.

The genome-wide mean DNA methylation levels  are inferred from the estimated *p*_*0*_ and *p*_*1*_ (Additional file 1). These are highly similar to, but slightly lower than, the fractional methylation levels ( in Table 2). Intuitively, because the non-conversion rate (*p*_*0*_) is substantial and on par with the mean methylation levels (Table 2), the fractional methylation levels at the face value could over-estimate the actual methylation levels. On the other hand, the fact that there may exist substantial levels of over-conversion (1-*p*_*1*_) indicates that ignoring the effect of over-conversion can lead to under-estimate the overall methylation levels. For instance, if we assume *p*_*1*_ = 0.95 (near perfect conversion), the estimated global methylation level  is much lower than fractional methylation (Table 2).

It is interesting to note that in the human data, the rate of over-conversion (1-*p*_*1*_) is much lower than in the honey bee data. Nevertheless, due to the over-conversion effectively under-estimating the actual methylation levels, the observed fractional methylation levels may be underestimates of the true methylation levels in the human genome. Again, if we assume a better over-conversion rate, the estimated global methylation level is closer to the observed fractional methylation levels (Table 2). Additional file 1 includes more detailed discussions on how these errors can affect estimation of DNA methylation differently in sparsely and heavily methylated genomes.

We then evaluated posterior odds of each site to classify each site as methylated or non-methylated. Local information is obtained from 3 kb adjacent to the focal CpG site (1.5 kb on each side), and the weight parameter used is 0.5. The numbers of methylated and non-methylated CpGs are shown in Table 2. In honeybee samples, Bis-Class detects on average 10% more methylated CpGs compared to the binomial method (Table 2). To determine whether this increase is due to high false positives or due to the improved inference, we investigated the difference between callings provided by the binomial method and the Bis-class methods further by several different approaches.

**Figure 5:**
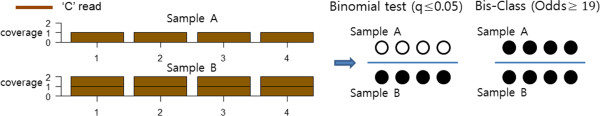
**The GB 16479 locus exhibits qualitatively identical information yet opposite methylation calling under the binomial method**. Data are from unpublished methylC-seq experiments of two honey bee individuals from the Yi lab, and are available upon request. All reads mapped to the four CpGs are ‘C’ reads (indicating 100% methylation). However, the binomial method provides a different methylation calls for these two samples. Specifically, the binomial calls all CpGs in the sample A as non-methylated (white dots), and all CpGs in the sample B as methylated (black dots). Bis-Class correctly identifies identical methylation features in the two replicates.

**Figure 6:**
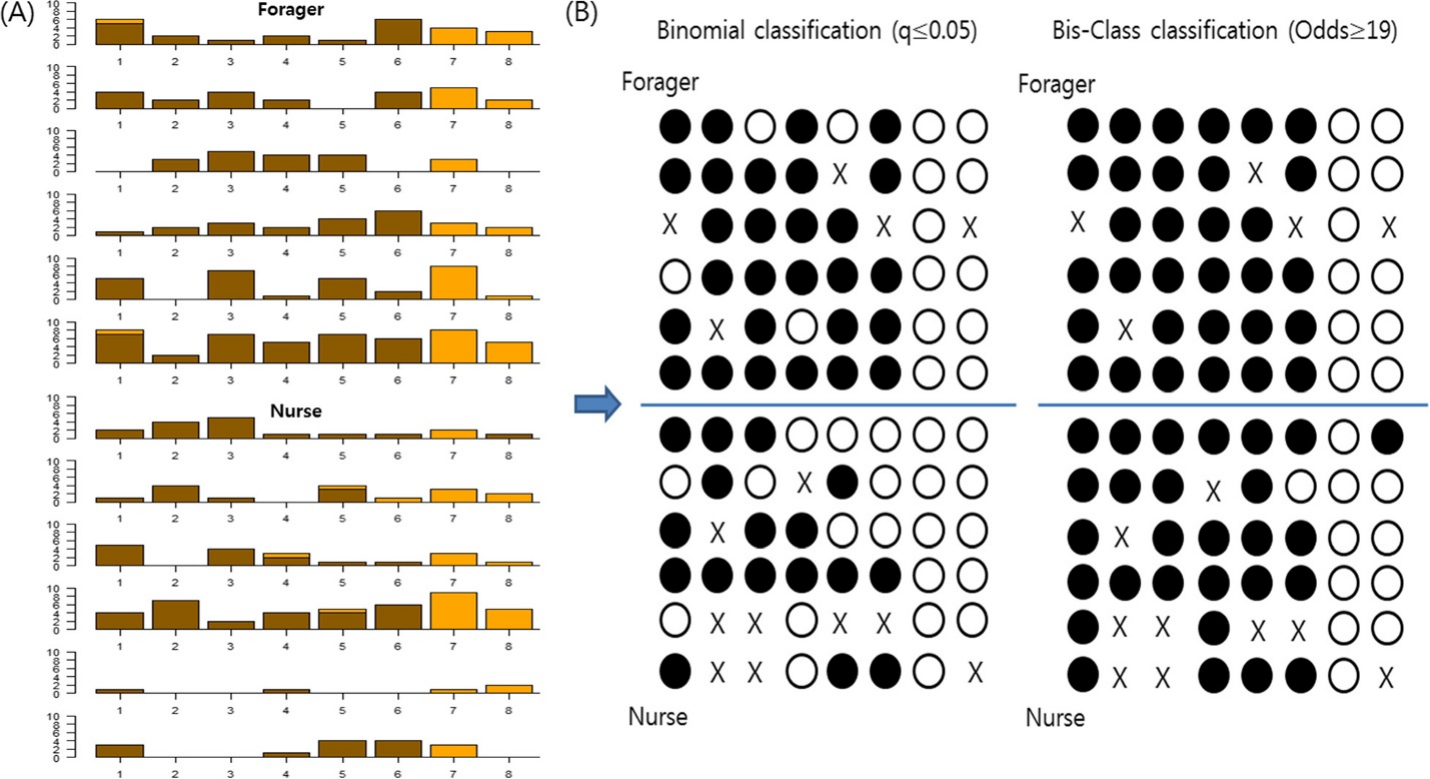
**Contrasting methylation**-**calling results of the GB 13135 locus in Herb et al. [**[7]**] data by the two methods.** **(A)** The numbers of ‘C’ reads (brown) and ‘T’ reads (orange) in 8 CpG positions of GB-13135. **(B)** Classification results following the binomial method (q-value < 0.05) and the Bis-Class method (Odds ≥ 19). CpGs classified as methylated are shown as black dots and those classified as non-methylated are shown as white dots. Sites with no read are marked as X. Bis-Class provides results that are more consistent across the biological replicates.

First, we found that many of these mCpGs detected by Bis-class are sites that are covered by a single C read that occur in highly methylated regions (Additional file 4). This improved detection is because while the binomial method cannot recognize any mCpGs covered by only a single read (e.g., Additional file 4), Bis-Class can provide methylation calling if that position occurs near other methylated CpGs. We demonstrate this property using two examples recovered from the data. The first example is the gene (GB-16479) from two honey bee MethylC-seq data sets (Figure 5). In this data, four cytosines cluster in a region with high overall methylation levels (the fractional methylation level of a 1000 bps encompassing these four sites is ~ 0.9 in both samples). In sample A, the four cytosines were covered by only single reads, all ‘C’s. In sample B, the same four cytosines were covered by two ‘C’ reads. The binomial method calls all cytosines in the first sample as ‘unmethylated’, while calling all four cytosines in the second sample as ‘methylated’. This example demonstrates the pitfalls of the binomial method clearly: two samples with exactly same qualitative information (100% ‘C’ reads in both cases) are classified as opposite directions due to the low sample size. Bis-Class, on the other hand, classified all four cytosines as ‘methylated’ for both cases. In the second example (Figure 6), we show the distribution of reads mapped to the locus GB 13135 in Herb et al. [7]. There are twelve samples in this data (six forager bees and six nurse bees). In the Forager 1 sample, the third and fifth positions are covered by single C reads. binomial method will call these as non-methylated (Figure 6B). However, since these sites occur in a heavily methylated region, Bis-Class calls both of these sites as methylated (Figure 6B). In other samples, these sites are covered by more than one read. For example, in the Forager 6 sample, both positions third and five are covered by seven C reads, and consequently called as methylated CpGs. The similarity between different biological replicates indicates that using local information improves methylation-calling accuracy. FDR-corrected q-values and posterior odds for each position of this locus are provided in the Additional file 5.

Second, we did the following experiments to directly assess the difference between the binomial method and Bis-Class when the numbers of reads is reduced. We assumed that we could distinguish methylated and non-methylated positions in coverage-rich CpG sites. We selected CpGs with over 7 coverage from the honey bee scaffold 1.1. There were 9300 CpGs that satisfied this criterion. For these coverage-rich sites, we considered those with < 10% ‘C’ reads as unmethylated, and > 30% ‘C’ reads as methylated. We then generated a new methyl-seq data set by randomly selecting only a single read from these sites, thereby artificially reducing the coverage. We then used the binomial method and Bis-Class for methylation calling. Since we have information on the true methylation status, we can directly assess the false positives and false negatives from this experiment. We also performed the same experiments for the coverages of two and three reads. Each experiment was repeated 100 times. The results of these analyses, shown in Additional file 6, demonstrate that Bis-Class is superior in these low coverage sites in the real data.

**Figure 7:**
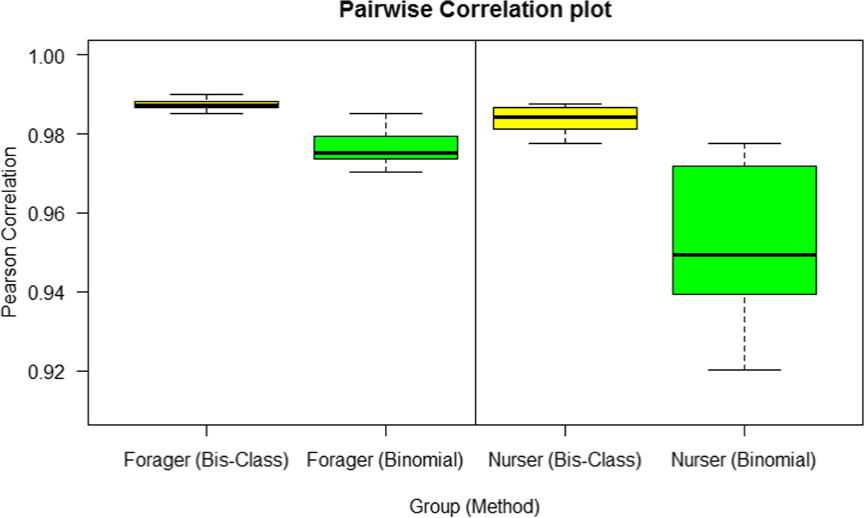
**Correlations between biological replicates are higher in the Bis**-**Class calling compared to the binomial calling.** The left panel represents the pairwise correlations between the methylation statuses of biological replicates in the forager samples from Herb et al. [7] data. The right panel represents the pairwise correlations between the nurse samples from the same study.

Third, we examined biological consistency across different methylC-seq data sets. We compared the calling results across the biological replicates offered by Herb et al. [7]. Bis-Class yields methylation callings that are more consistent among biological replicates. First, the coefficient of variation (CV) of methylated CpG counts in 12 samples from Bis-Class (0.067) is less than half of the CV from the binomial method (0.150). Second, we calculated pairwise correlations of gene methylation levels between samples in each subtype (foragers and nurses). Correlations between individuals are much greater for Bis-Class than those via the binomial method (Figure 7). Based on the biological facts that methylation pattern are similar between individuals in the same species (e.g., Figure 2E), the observed higher correlations implies more realistic classification of DNA methylation via Bis-Class. We also note that in the binomial method, pairwise correlations are highly sensitive to the coverage levels. Specifically, nurse samples have more variable coverage than forager samples (Table 1), and the calling via binomial method is highly variable, in contrast to the more stable methylation calling from Bis-Class.

## Conclusions

The development of the methylC-seq method has propelled genome-wide evaluation of DNA methylation in diverse genomes across the tree of life. Due to the next-generation sequencing nature of methylC-seq, the information content at each position varies greatly. Given such constraints, statistical methods that can perform robustly, even when sequence coverage is low, are desired. The existing binomial method is prone to errors in low coverage sites, particularly in sparsely methylated genomes. Our approach solves this problem by explicitly incorporating local DNA methylation levels in a Bayesian framework. This is based upon the observation that methylated sites are locally clustered. By utilizing both global and local methylation information, we can obtain more biologically consistent and relevant information. Bis-Class is particularly well-suited in the analyses of sparsely methylated genomes such as insect genomes.

### Availability of supporting data

All datasets used in our study can be found in Gene Expression Omnibus (GEO) site (http://www.ncbi.nlm.nih.gov/geo) using below accession IDs.

Honeybee DNA methylation data from reference [7]: GSE36650.

Honeybee DNA methylation data from reference [8]: GSE56399.

Human brain DNA methylation data from reference [15]: GSE37202.

Arabidopsis DNA methylation data from reference [29]: GSM276809.

## Electronic supplementary material

Additional file 1: **Estimation of error rates using Expectation-Maximization (EM) algorithm.** (DOCX 28 KB)

Additional file 2: **Using high-confidence CpG sites (coverage ≥7) and sampling one read for each site, we examined the AUC, sensitivity, and 1-specificity of different kernels and weight factors.** The results indicate that the three kernels tried (Triangle, Gaussian, and Laplace) provide similarly high sensitivity and acceptable 1-specificity. Gaussian kernel performs slightly worse with respect to sensitivity. (DOCX 2 MB)

Additional file 3: **Comparison of the AUC measures in simulated data sets.** Parameter settings of the simulation are identical with those in the Figures 3 and 4 in the main text. AUC is generally higher for the Bis-Class compared to the Binomial method. (DOCX 2 MB)

Additional file 4: **Histogram of mCpG counts detected using the Bis-Class and the Binomial method.** Red and blue bars are the results from the Bis-Class and the Binomial method, respectively. X-axis indicates the coverage of each site and the Y-axis indicates the sum of methylated CpG counts in the 12 samples in Herb et al. [7]. (DOCX 1 MB)

Additional file 5: **q-values and odds of 12 honeybee samples in GB-13135 which is displayed in Figure** 6 **.** (DOCX 25 KB)

Additional file 6: **Comparison of three accuracy measures (AUC, sensitivity and specificity) evaluated from the confirmation analyses.** We used high coverage CpG sites and then reduced their coverages to 1 and analyzed how well each method performs. using reduced coverage honeybee data: the X-axis indicates the number of reads. Definitions of sensitivity and specificity are identical with those used in the Figures 3 and 4. Violet bars imply results of the Binomial method and green bars imply results of the Bis-Class. (DOCX 170 KB)
