# Supplementary material for: Bis-class: a new classification tool of methylation status using bayes classifier and local methylation information
Source: BMC Genomics. 2014 Jul 18;15(1):608. doi: 10.1186/1471-2164-15-608 (PMC4117951; doi:10.1186/1471-2164-15-608)
Supplement: Supplementary file 1 — Additional file 1: Estimation of error rates using Expectation-Maximization (EM) algorithm. (DOCX 28 KB) [file 12864_2014_6293_MOESM1_ESM.docx]

**Additional File 1**

**Estimation of error rates using Expectation-Maximization (EM) algorithm**

The parameters $p_{0}$ and $p_{1}$ are inferred from data using the Expectation-Maximization (EM) algorithm [21]. Because $p_{0}$ and $p_{1}$ are probabilities of emerging ‘C’ read in a non-methylated site and a methylated site, respectively; latent status for them are given as non-methylated (M=*nm*) or methylated (M=*m*). For convenience, 0 and 1 denote *nm* and *m* for methylation status, respectively. Expected complete log-likelihood for $N^{'}$ selected samples is then given as

$Q\left( \theta| \hat{\theta}_{t} \right)=E\left[ log\prod_{i=1}^{N^{'}} \left[ \hat{\pi}_{1,t}\times{\hat{p}_{1,t}}^{C_{i}}\times\left( 1-\hat{p}_{1,t} \right)^{T_{i}} \right]^{M_{i}}\left[ \hat{\pi}_{0,t}\times{\hat{p}_{0,t}}^{C_{i}}\times\left( 1-\hat{p}_{0,t} \right)^{T_{i}} \right]^{1-M_{i}} \right]$. (7)

$\hat{\theta}_{t}$ is a set of parameter obtatined $t^{th}$ iteration and $\hat{\pi}_{0t}$, $\hat{p}_{0t}$ and$\hat{p}_{1t}$ are its components. From this equation, we can calculate probability of being methylated as

$\hat{\mu}_{i,t}=E\left[ M_{i} | C_{i},T_{i}, \hat{\theta}_{t} \right]=p\left( M_{i}=1 | C_{i},T_{i}, \hat{\theta}_{t} \right)=\frac{\hat{\pi}_{1,t}\times{\hat{p}_{1,t}}^{C_{i}}\times\left( 1-\hat{p}_{1,t} \right)^{T_{i}}}{\hat{\pi}_{1,t}\times{\hat{p}_{1,t}}^{C_{i}}\times\left( 1-\hat{p}_{1,t} \right)^{T_{i}}+\hat{\pi}_{0,t}\times{\hat{p}_{0,t}}^{C_{i}}\times\left( 1-\hat{p}_{0,t} \right)^{T_{i}}}$. (8)

In the maximization step, $\hat{\theta}_{t+1}$ can be obtained by maximizing $Q\left( \theta| \hat{\theta}_{t} \right)$ and the solutions are given as

$\hat{\pi}_{1,t+1}=\frac{1}{N^{'}}\sum_{i} \hat{\mu}_{i,t}$ ,$\hat{p}_{0,t+1}=\frac{\sum_{i} \hat{\mu}_{i,t}\times C_{i}}{\sum_{i} \hat{\mu}_{i,t}}$, $\hat{p}_{0,t+1}=\frac{\sum_{i} (1-\hat{\mu}_{i,t})\times C_{i}}{\sum_{i} {(1-\hat{\mu}}_{i,t})}$. We iterated this process until change of $\hat{\theta}_{t}$ is small enough to be regarded as conversion. Then the converged estimates are maximum likelihood estimate of $p_{0}$ and $p_{1}$. In order to obtain independent samples for constructing likelihood function used in EM, we divided the whole genome by 10kb windows and select a site for each window by the result from Figure 2C. We repeated this process 100 times and calculated median of the results to obtain final estimates of $p_{0}$ and $p_{1}$. If we already know $\hat{p}_{0}$ from the experiment, we may fix $p_{0}$ in the EM algorithm and maximize likelihood only to estimate of $p_{1}$.

**Global DNA Methylation Level**

In this section we describe the estimation process for the global methylation levels, ${\hat{\pi}_{1}}^{G}$ and ${\hat{\pi}_{0}}^{G}$ which are the estimates of proportion of methylated and non-methylated sites in the whole methylome. We define $C_{i}$ as number of C reads assigned to an $i^{th}$ site, $T_{i}$ as the number of T reads assigned to the $i_{th}$ site. The total number of reads in the $i^{th}$site of a sample $L_{i}$ is then $C_{i}$*+*$T_{i}$. Then the proportion of cytosine read in $i^{th}$ site, $F_{i}=C_{i}/L_{i}$, is equivalent to the widely used ‘fractional methylation’ measure [13-15]. Therefore, for any *i*, expectation of $F_{i}$ can be estimated as:

$E(F_{i})=\pi_{1}$ $\times E(F_{i}|M=m)$+$\pi_{0}$ $\times E\left( F_{i} | M=nm \right)=\pi_{1}\times p_{1}+\pi_{0}\times p_{0}$. (9)

Using the method of moments and adjusting for the total read count $L_{i}$ to impose more confidence to deep coverage sites, the estimate of $E(F)$ across whole genome will be $(1/\sum_{i=1}^{N} \sqrt{L_{i}})\times(\sum_{i=1}^{N} {\sqrt{L_{i}}\times F}_{i})$.

From equation (9) and $\pi_{1}=1-\pi_{0}$,$\hat{\pi}_{1}$for global methylome, denoted by ${\hat{\pi}_{1}}^{G}$, is as follows:

$(\hat{E(F)}-\hat{p}_{0})/(\hat{p}_{1}-\hat{p}_{0}).$ (10)

In case of local methylation level, $E(F)$ is estimated as $(1/\sum_{i=1}^{N} \sqrt{L_{i}}\times K\left( d_{k} \right))\times(\sum_{i=1}^{N} {\sqrt{L_{i}}\times F}_{i}\times K\left( d_{k} \right))$ which additionally adopts kernel weight $K\left( d_{k} \right)$.

**Effects of Non-Conversion and Over-Conversion for Estimating Global Methylation Levels in Sparsely and Heavily Methylated Genomes**

The estimate of global methylation level is, as shown in the equation (10), affected by both i) the numerator term, which is the difference between the global methylation level and the non-conversion rate (false positives) and ii) the denominator term, which is the difference between over-conversion (false negatives) and non-conversion. In sparsely methylated genomes, the non-conversion rate, which affects the numerator significantly, will have a larger influence. In heavily methylated genomes, because the non-conversion rate is negligible compared to the overall methylation levels, the numerator will not change much by the correction, and the overall methylation levels will be more influenced by the over-conversion rate in the denominator.

For example, In the case of honey bee data in Table 2, mean methylation level $\hat{E(F)}$ (=0.0033) is only about twice that of $\hat{p}_{0}$ (=0.0015) and therefore the numerator $\hat{E(F)}-\hat{p}_{0}$ is reduced almost by half to 0.0018. Further dividing this further by $\hat{p}_{1}-\hat{p}_{0}$(=0.65) leads to a smaller estimate of global methylation level compared to the $\hat{E(F)}$, especially when $\hat{p}_{1}$ is close to one. However, in the human data, $\hat{E\left( F \right)}$(=0.8064) is about 80 times bigger than $\hat{p}_{0}$(=0.01), and subtracting $\hat{p}_{0}$ from $\hat{E(F)}$ does not make big difference. Further dividing this number further by $\hat{p}_{1}-\hat{p}_{0}$(=0.93) makes $\hat{\pi}_{1}$ even bigger than $\hat{E(F)}$. In summary, if $\hat{E(F)}$ is big compared to $\hat{p}_{0}$ and $\hat{p}_{1}-\hat{p}_{0}$ is small, $\hat{\pi}_{1}$ will be generally bigger than $\hat{E(F)}$.

Biologically speaking, in the honey bee data, the non-conversion error rate is similar to the global methylation level, thus overall inflate the methylation levels. Correcting for the errors will thus reduce the estimated methylation level. In the human data however, the non-conversion rate error is generally negligible compared to the actual methylation levels. The effect of over-conversion, even though lower than for the honey bee data, generally deflate the global methylation levels. Correcting for errors will thus increase the methylation levels.

**Kolmogorov-Smirnov test for testing spatial correlation**

Figure 2C shows a quantile-quantile (Q-Q) plot to determine the physical distance required for all CpGs to be mutually uncorrelated. We selected a CpG for each window and calculated C/(C+T) for all selected CpGs. The methylation fraction of $i^{th}$ selected CpG is denoted as $F_{i}$ and i= 1, 2, …, N. Then we calculated $\tilde{F}_{i}=F_{i}-\frac{1}{N}\sum F_{i}$, which denotes residual of $F_{i}$. Since methylation level does not follow normal distribution, the Durbin-Watson test, which is popularly used in regression analysis for test of independence of residuals, is impractical. Instead, we used nonparametric procedures. First, we extracted the sign of $\tilde{F}_{i}$ and then tested whether positive loci are uniformly distributed via the Kolmogorov-Smirnov test. For a fixed window size, we repeated the procedure 1,000 times and compared the obtained p-values with the theoretical p-values, based on the null distribution. The distribution of observed p-values becomes concordant with the distribution of theoretical p-values as window size increases, and we can determine the window size which imposes mutual independence on all selected CpGs from these results.
