# Supplementary material for: Bis-class: a new classification tool of methylation status using bayes classifier and local methylation information
Source: BMC Genomics. 2014 Jul 18;15(1):608. doi: 10.1186/1471-2164-15-608 (PMC4117951; doi:10.1186/1471-2164-15-608)

**Additional File 2**

Using high-confidence CpG sites (coverage $\geq$7) and sampling one read for each site, we examined the AUC, sensitivity, and 1-specificity of different kernels and weight factors. The results indicate that the three kernels tried (Triangle, Gaussian, and Laplace) provide similarly high sensitivity and acceptable 1-specificity. Gaussian kernel performs slightly worse with respect to sensitivity.


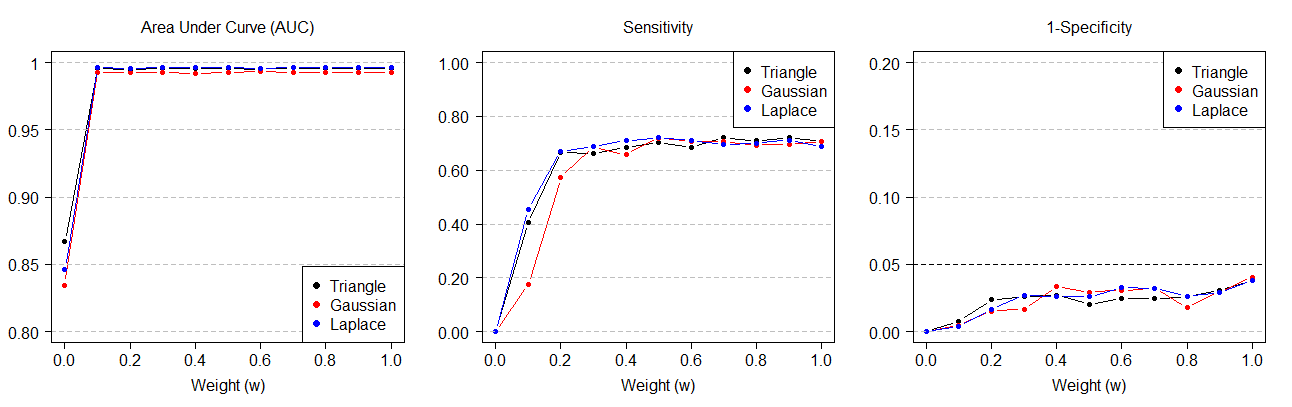

Supplement: Supplementary file 2 — Additional file 2: Using high-confidence CpG sites (coverage ≥7) and sampling one read for each site, we examined the AUC, sensitivity, and 1-specificity of different kernels and weight factors. The results indicate that the three kernels tried (Triangle, Gaussian, and Laplace) provide similarly high sensitivity and acceptable 1-specificity. Gaussian kernel performs slightly worse with respect to sensitivity. (DOCX 2 MB) [file 12864_2014_6293_MOESM2_ESM.docx]
