# Supplementary material for: Bis-class: a new classification tool of methylation status using bayes classifier and local methylation information
Source: BMC Genomics. 2014 Jul 18;15(1):608. doi: 10.1186/1471-2164-15-608 (PMC4117951; doi:10.1186/1471-2164-15-608)

**Additional File 3.** Comparison of the AUC measures in simulated data sets. Parameter settings of the simulation are identical with those in the Figures 3 and 4 in the main text. AUC is generally higher for the Bis-Class compared to the Binomial method.


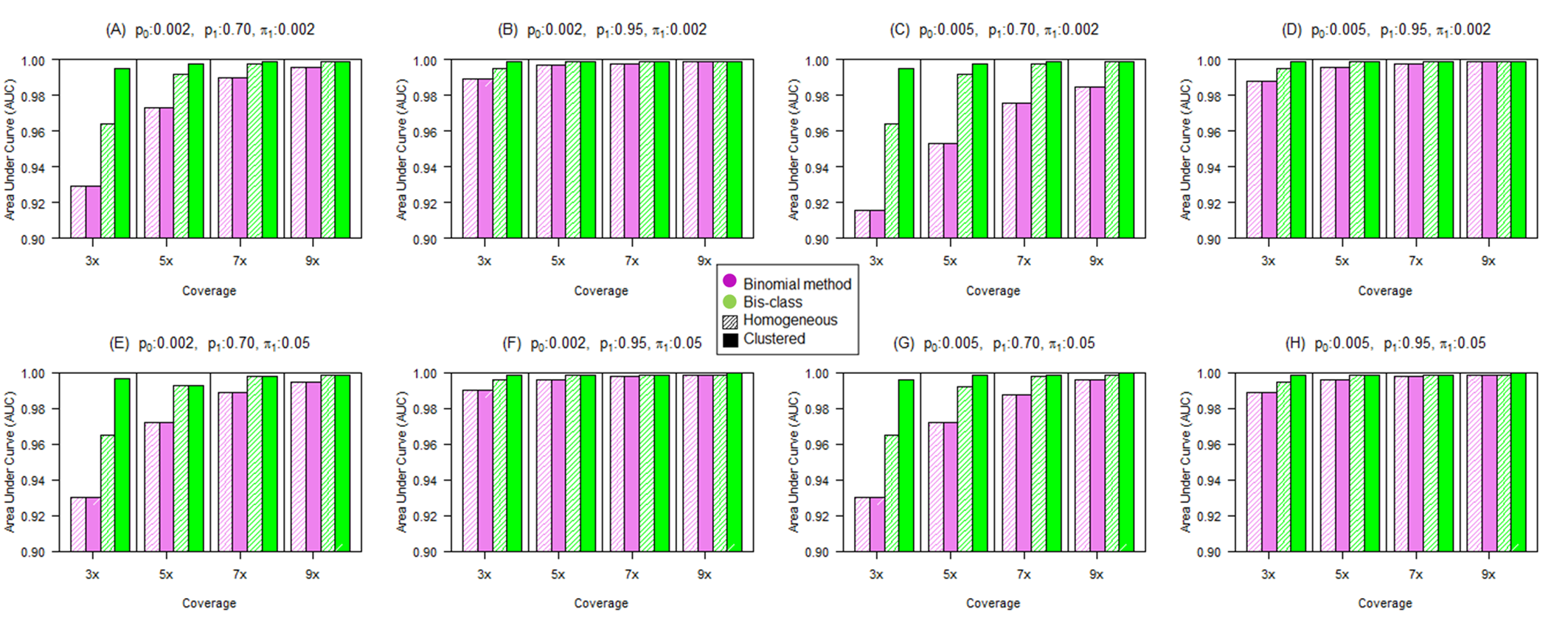

Supplement: Supplementary file 3 — Additional file 3: Comparison of the AUC measures in simulated data sets. Parameter settings of the simulation are identical with those in the Figures 3 and 4 in the main text. AUC is generally higher for the Bis-Class compared to the Binomial method. (DOCX 2 MB) [file 12864_2014_6293_MOESM3_ESM.docx]
