# Supplementary material for: Bis-class: a new classification tool of methylation status using bayes classifier and local methylation information
Source: BMC Genomics. 2014 Jul 18;15(1):608. doi: 10.1186/1471-2164-15-608 (PMC4117951; doi:10.1186/1471-2164-15-608)

**Additional File 4.** Histogram of mCpG counts detected using the Bis-Class and the Binomial method. Red and blue bars are the results from the Bis-Class and the Binomial method, respectively. X-axis indicates the coverage of each site and the Y-axis indicates the sum of methylated CpG counts in the 12 samples in Herb et al. [7].


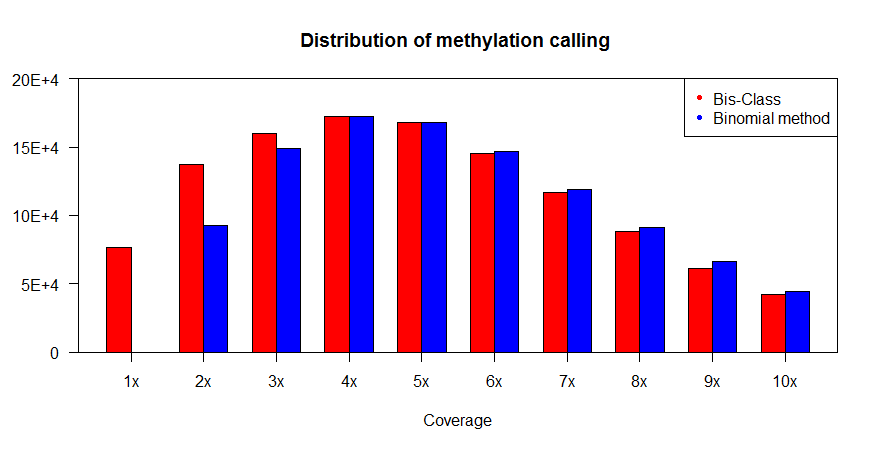

Supplement: Supplementary file 4 — Additional file 4: Histogram of mCpG counts detected using the Bis-Class and the Binomial method. Red and blue bars are the results from the Bis-Class and the Binomial method, respectively. X-axis indicates the coverage of each site and the Y-axis indicates the sum of methylated CpG counts in the 12 samples in Herb et al. [7]. (DOCX 1 MB) [file 12864_2014_6293_MOESM4_ESM.docx]
