# Supplementary material for: Bis-class: a new classification tool of methylation status using bayes classifier and local methylation information
Source: BMC Genomics. 2014 Jul 18;15(1):608. doi: 10.1186/1471-2164-15-608 (PMC4117951; doi:10.1186/1471-2164-15-608)
Supplement: Supplementary file 5 — Additional file 5: q-values and odds of 12 honeybee samples in GB-13135 which is displayed in Figure 6 . (DOCX 25 KB) [file 12864_2014_6293_MOESM5_ESM.docx]

**Additional File 5.** q-values and odds of 12 honeybee samples in GB-13135 which is displayed in Figure 6.

| Position  Sample | | 1 | 2 | 3 | 4 | 5 | 6 | 7 | 8 |
| --- | --- | --- | --- | --- | --- | --- | --- | --- | --- |
| SRR445767 | q | 2.63E-11 | 4.59E-4 | 0.22 | 4.59E-4 | 0.22 | 0 | 1 | 1 |
|  | Odds | 3.01E+12 | 1.04E+05 | 2.20E+02 | 9.54E+04 | 1.97E+02 | 3.15E+15 | 5.86E-03 | 1.74E-02 |
| SRR445768 | q | 1.77E-9 | 4.00E-4 | 1.77E-9 | 0.0004 | NA | 1.77E-9 | 1 | 1 |
|  | Odds | 1.25E+10 | 7.07E+04 | 1.08E+10 | 6.09E+04 | NA | 8.99E+09 | 1.65E-03 | 3.66E-02 |
| SRR445769 | q | NA | 7.74E-7 | 3.47E-12 | 1.6E-9 | 1.6E-9 | NA | 1 | NA |
|  | Odds | NA | 6.34E+7 | 1.23E+13 | 2.64E+10 | 2.33E+10 | NA | 0.0207 | NA |
| SRR445770 | q | 0.200 | 3.82E-4 | 7.23E-07 | 3.82e-4 | 1.45E-09 | 0 | 1 | 1 |
|  | Odds | 216.13 | 9.66E+4 | 3.84E+7 | 8.52E+4 | 1.50E+10 | 3.25E+15 | 0.0167 | 0.0467 |
| SRR445771 | q | 2.27E-12 | NA | 0 | 0.197775 | 2.27E-12 | 0.000357 | 1 | 1 |
|  | Odds | 1.34E+13 | NA | 2.79E+18 | 255.2 | 1.13E+13 | 108110 | 7.77E-05 | 0.1639 |
| SRR445773 | q | 0 | 3.38E-4 | 0 | 1.75E-12 | 0 | 0 | 1 | 1 |
|  | Odds | 2.50E+18 | 1E+5 | 2.25E+18 | 1.04E+13 | 1.97E+18 | 4.23E+15 | 8.46E-05 | 2.06E-3 |
| SRR445774 | q | 5.8E-4 | 3.73E-09 | 1.05E-11 | 0.240 | 0.240 | 0.240 | 1 | 0.240 |
|  | Odds | 1.26E+5 | 2.55E+10 | 1.03E+13 | 252.28 | 220 | 219.81 | 0.0499 | 206.43 |
| SRR445775 | q | 0.216 | 1.77E-9 | 0.216 | NA | 3.18E-6 | 1 | 1 | 1 |
|  | Odds | 149.29 | 1.11E+10 | 124.91 | NA | 6.88E+6 | 0.097517 | 1.13E-2 | 3.23E-2 |
| SRR445776 | q | 5.71E-12 | NA | 2.37E-9 | 0.001332 | 0.223 | 0.223 | 1 | 1 |
|  | Odds | 5.06E+12 | NA | 1.06E+10 | 2.18E+4 | 122.49 | 122.37 | 0.0129 | 0.103 |
| SRR445777 | q | 1.67E-09 | 0 | 4.22E-4 | 1.67E-09 | 7.66E-9 | 0 | 1 | 1 |
|  | Odds | 1.56E+10 | 1.26E+18 | 7.35E+4 | 1.36E+10 | 4.16E+09 | 2.21E+15 | 2.40E-05 | 0.00169 |
| SRR445778 | q | 0.190 | NA | NA | 0.190 | NA | NA | 1 | 1 |
|  | Odds | 169.12 | NA | NA | 137.63 | NA | NA | 0.0899 | 0.0316 |
| SRR445799 | q | 8.98E-7 | NA | NA | 0.215 | 1.92E-9 | 1.92E-9 | 1 | NA |
|  | Odds | 3.82E+7 | NA | NA | 182.67 | 1.21E+10 | 1.21E+10 | 0.01557 | NA |
