# Supplementary material for: Bis-class: a new classification tool of methylation status using bayes classifier and local methylation information
Source: BMC Genomics. 2014 Jul 18;15(1):608. doi: 10.1186/1471-2164-15-608 (PMC4117951; doi:10.1186/1471-2164-15-608)

**Additional File 6.** Comparison of three accuracy measures (AUC, sensitivity and specificity) evaluated from the confirmation analyses. We used high coverage CpG sites and then reduced their coverages to 1 and analyzed how well each method performs. using reduced coverage honeybee data: the X-axis indicates the number of reads. Definitions of sensitivity and specificity are identical with those used in the Figures 3 and 4. Violet bars imply results of the Binomial method and green bars imply results of the Bis-Class.


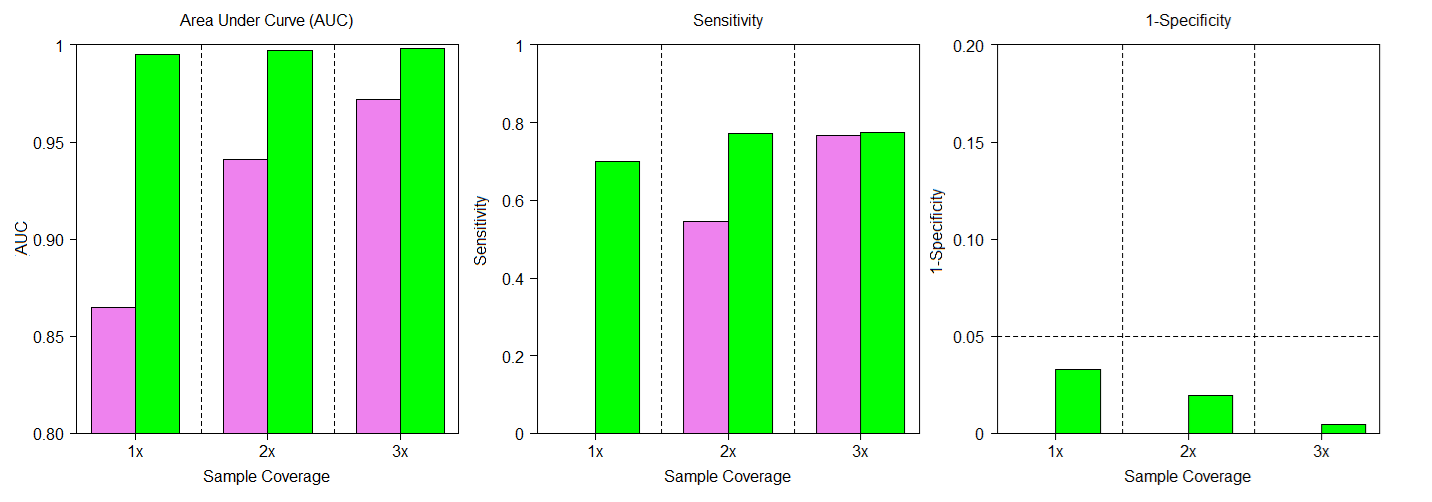

Supplement: Supplementary file 6 — Additional file 6: Comparison of three accuracy measures (AUC, sensitivity and specificity) evaluated from the confirmation analyses. We used high coverage CpG sites and then reduced their coverages to 1 and analyzed how well each method performs. using reduced coverage honeybee data: the X-axis indicates the number of reads. Definitions of sensitivity and specificity are identical with those used in the Figures 3 and 4. Violet bars imply results of the Binomial method and green bars imply results of the Bis-Class. (DOCX 170 KB) [file 12864_2014_6293_MOESM6_ESM.docx]
